# Plasma Carotenoids, Tocopherols, and Retinol in the Age-Stratified (35–74 Years) General Population: A Cross-Sectional Study in Six European Countries

**DOI:** 10.3390/nu8100614

**Published:** 2016-09-30

**Authors:** Wolfgang Stuetz, Daniela Weber, Martijn E. T. Dollé, Eugène Jansen, Beatrix Grubeck-Loebenstein, Simone Fiegl, Olivier Toussaint, Juergen Bernhardt, Efstathios S. Gonos, Claudio Franceschi, Ewa Sikora, María Moreno-Villanueva, Nicolle Breusing, Tilman Grune, Alexander Bürkle

**Affiliations:** 1 Institute of Biological Chemistry and Nutrition, University of Hohenheim, Stuttgart 70599, Germany; Wolfgang.Stuetz@uni-hohenheim.de (W.S.); breusing@uni-hohenheim.de (N.B.); 2 Institute of Nutrition, Friedrich-Schiller-University, Jena 07743, Germany; 3 Department of Molecular Toxicology, German Institute of Human Nutrition Potsdam-Rehbruecke (DIfE), Nuthetal 14558, Germany; scientific.director@dife.de; 4 National Institute of Public Health and the Environment (RIVM), BA Bilthoven 3721, The Netherlands; Martijn.Dolle@rivm.nl (M.E.T.D.); eugene.jansen@rivm.nl (E.J.); 5 Institute for Biomedical Aging Research, Leipold-Franzens-University, Innsbruck 6020, Austria; beatrix.grubeck@uibk.ac.at; 6 Institute for Nutritional Sciences and Physiology, University for Health Sciences, Hall in Tirol 6060, Austria; simone.fiegl@umit.at; 7 Unit of Cellular Biochemistry and Biology, University of Namur, Namur 5000, Belgium; olivier.toussaint@fundp.ac.be; 8 BioTeSys GmbH, Esslingen 73728, Germany; j.bernhardt@biotesys.de; 9 Institute of Biological Research and Biotechnology, National Hellenic Research Foundation (NHRF), Athens 11635, Greece; sgonos@eie.gr; 10 Department of Experimental Pathology, University of Bologna, Bologna 40126, Italy; claudio.franceschi@unibo.it; 11 Nencki Institute of Experimental Biology, Polish Academy of Sciences, Warsaw 02-093, Poland; e.sikora@nencki.gov.pl; 12 Molecular Toxicology, Department of Biology, University of Konstanz, Konstanz 78457, Germany; maria.moreno-villanueva@uni-konstanz.de (M.M.-V.); alexander.buerkle@uni-konstanz.de (A.B.); 13 Department of Applied Nutritional Science/Dietetics, Institute of Nutritional Medicine, University of Hohenheim, Stuttgart 70599, Germany; 14 German Center for Diabetes Research (DZD), Munich-Neuherberg 85764, Germany; 15 German Center for Cardiovascular Research (DZHK), Berlin 13357, Germany; 16 NutriAct-Competence Cluster Nutrition Research Berlin-Potsdam, Nuthetal 14458, Germany

**Keywords:** carotenoids, plasma, age, Europe, micronutrients, lycopene, retinol, tocopherols

## Abstract

Blood micronutrient status may change with age. We analyzed plasma carotenoids, α-/γ-tocopherol, and retinol and their associations with age, demographic characteristics, and dietary habits (assessed by a short food frequency questionnaire) in a cross-sectional study of 2118 women and men (age-stratified from 35 to 74 years) of the general population from six European countries. Higher age was associated with lower lycopene and α-/β-carotene and higher β-cryptoxanthin, lutein, zeaxanthin, α-/γ-tocopherol, and retinol levels. Significant correlations with age were observed for lycopene (*r* = −0.248), α-tocopherol (*r* = 0.208), α-carotene (*r* = −0.112), and β-cryptoxanthin (*r* = 0.125; all *p* < 0.001). Age was inversely associated with lycopene (−6.5% per five-year age increase) and this association remained in the multiple regression model with the significant predictors (covariables) being country, season, cholesterol, gender, smoking status, body mass index (BMI (kg/m^2^)), and dietary habits. The positive association of α-tocopherol with age remained when all covariates including cholesterol and use of vitamin supplements were included (1.7% vs. 2.4% per five-year age increase). The association of higher β-cryptoxanthin with higher age was no longer statistically significant after adjustment for fruit consumption, whereas the inverse association of α-carotene with age remained in the fully adjusted multivariable model (−4.8% vs. −3.8% per five-year age increase). We conclude from our study that age is an independent predictor of plasma lycopene, α-tocopherol, and α-carotene.

## 1. Introduction

Epidemiological studies in the United States and in Europe suggest a lower risk of major chronic age-related diseases (cardiovascular diseases, diabetes, cancer) and an increased life expectancy with diets high in fruit and vegetables, and consequently higher blood concentrations of vitamins and carotenoids [1,2,3,4,5,6,7]. Some micronutrients exert antioxidant activities, which are responsible for some of their physiological functions, e.g., the anti-inflammatory properties of carotenoids in quenching singlet oxygen and peroxyl radicals [8,9] and the membrane-stabilizing effects of vitamin E [10]. Furthermore, micronutrients are key players in cellular homeostasis and in general physiology. A healthy diet and lifestyle are simple, safe, cheap, and effective ways to increase health span, i.e., the functional and disease-free period of life. Therefore, the influence of a carotenoid- and antioxidant-rich diet on the aging process is of increasing interest since life expectancy is steadily increasing in Western countries.

Blood concentrations of micronutrients such as carotenoids and vitamins vary due to dietary habits, season, country, smoking status, and gender and may also be affected by age [11,12,13,14,15,16,17,18,19]. Previous medium- and large-scale studies have already revealed associations of blood micronutrients with age. These studies have not always covered a stratified and wide age distribution, or did not analyze micronutrients in both genders. Furthermore, most studies have been carried out in the United States and few in Europe, but it is known that dietary habits differ strongly between these continents/regions. Therefore we measured plasma carotenoids, tocopherols, and retinol in healthy participants aged 35–74 years as part of the MARK-AGE study, a large-scale European multicenter study aiming to identify biomarkers of age and healthy aging [20]. The main objective of the present study was to elucidate the role of age, other demographic characteristics, and dietary habits on the plasma concentrations of carotenoids, tocopherols, and retinol in a large age-stratified group recruited from the general European population.

## 2. Materials and Methods

### 2.1. Study Population and Sample Collection

After the identification of a specific region (i.e., a small/medium-sized town near a participating study/recruiting center) having a sufficient number of eligible subjects, participants were recruited in seven different centers through various public platforms including radio advertising and newspaper articles near the centers. Inclusion criteria were age 35–74 years (both genders) and ability to give informed consent. In total, 2353 healthy male and female volunteers were recruited from the general population from seven European countries (Austria, Belgium, Finland, Germany, Greece, Italy, and Poland) for MARK-AGE in an age-stratified manner. The recruitment of the participants and the collection of anthropometric markers (weight, height), medical history (interviewed by trained field workers), and lifestyle characteristics took place between November 2008 and June 2012 at the following centers: Hall in Tirol and Innsbruck (Austria), Namur (Belgium), Esslingen (Germany), Athens and nearby regions (Greece), Bologna (Italy), Warsaw (Poland), and Tampere (Finland). Publications describing the project design, study population, and standard operating procedures have been recently published [20,21,22].

### 2.2. Ethics Statement and Field Procedure

Ethical clearance was given by the ethics committee of each of the centers. Furthermore, the study has retrospectively been registered at the German Clinical Trials Register (DRKS00007713). Subjects who reported seropositivity for HIV or hepatitis (HBV, HCV), who tested positive for HBV or HCV, or were being treated for cancer or receiving glucocorticoids were excluded from the study. Participants completed a comprehensive questionnaire on self-reported lifestyle characteristics and dietary intake. The questionnaire on lifestyle characteristics included information on smoking status (frequencies given as: Current smoker, former, never). The short non-quantitative dietary questionnaire consisted of a checklist of foods and beverages with a frequency response section. It did not contain portion sizes for meals but for beverages: the serving size for water, juice, soft drinks, beer, and wine was 200 mL (1 glass), whereas the serving size for other alcoholic drinks was 20 mL (1 shot). Participants were asked to write the average amount of consumed drinks (*n* glasses) in the most appropriate frequency category as *n* per day, *n* per week, *n* per month, or never; from this reported data we finally calculated the frequencies of consumed glasses: <1x/day, 1x/day, or ≥2x/day. The following frequencies were given in the questionnaire for participants to tick one box for each food item (fruit, vegetables, French fries, and meat) and use of vitamin supplements: ≥2x /day, 1x/day, 4–6x/week, 1–3x/week, 1–3x/month, never. The questionnaire has been shown to be repeatable and valid when compared to a detailed food frequency questionnaire [23]. Collected data were merged, saved, and managed by the central database at the Department of Biology at the University of Konstanz (Konstanz, Germany).

All nurses and technicians responsible for blood collection were trained together in a workshop prior to sample collection (blood, urine, buccal mucosa) to guarantee a standardized procedure. Venous blood was collected by venipuncture into vacutainers (Greiner Bio One cryotubes) in the morning after an overnight fast, processed within 3–5 h to obtain aliquots of whole blood, plasma, or serum (portioned into Eppendorf tubes coded with the primary subject code), which were immediately frozen at −80 °C until shipment (on dry ice) to the MARK-AGE Biobank located at the University of Hohenheim (Stuttgart, Germany). Blood samples were tested for hepatitis virus infection (HBV, HCV) before final inclusion into the study. Using a secondary subject code (SSC), all biological samples free of HBV/HCV were then processed by the Biobank and personal data were entered into the central database. SSC-coded plasma and serum aliquots were shipped (on dry ice) to the Institute of Nutrition, Friedrich-Schiller-University (Jena, Germany), and the National Institute of Public Health and the Environment (RIVM, Bilthoven, The Netherlands), respectively, where they were stored at −80 °C until analysis. Results from the SSC-coded samples were uploaded to the central database [21].

Out of a total of 2262 eligible subjects, 2207 subjects with available plasma samples and 2082 with simultaneously available serum samples remained for analyses of nutritional biomarkers (carotenoids, tocopherols and retinol) and cholesterol, respectively, after the exclusion of subjects who tested positive for hepatitis. For the present analyses, all 89 subjects from Finland were excluded due to the small sample size and only a few cases in the younger age groups (35–59 years) in comparison to the other centers. Six subjects who had just turned 75 years at the time of blood sampling were included in the highest age group of 70–74. Thus, a total of 2118 datasets of women and men from six different countries were assessed for the relationship of age with demographic characteristics, self-reported dietary intake, and plasma carotenoids, tocopherols, retinol, and cholesterol.

### 2.3. Laboratory Methods

The carotenoids lutein, zeaxanthin, β-cryptoxanthin, lycopene, and α-/β-carotene, α-/γ-tocopherol, and retinol in plasma were simultaneously determined by HPLC with UV and fluorescence detection as previously described [24]. In brief, plasma (40 µL) was extracted with ethanol/*n*-butanol (1:1, 200 µL) containing β-apo-8′-carotenal-methyloxime as an internal standard. After centrifugation (21,000× *g*, 15 min at 4 °C), 20 µL of the clear supernatant was analyzed on a Shimadzu Prominence HPLC (LC-20A) with chromatographic conditions, as previously described in detail [24]. Pure standard mixtures which were prepared and run as a sample were used for quantification. These standards were verified against serum pools with assigned values set against the Standard Reference Material (SRM 968c, NIST, Gaithersburg, MD, USA). For internal quality control, aliquots of a plasma pool run along within the > 30 batches gave inter-batch coefficients of variations (CVs) for carotenoids <8% (between 3.1% for α-carotene to 7.6% for lycopene), tocopherols <7% (4.1% for γ-tocopherol and 6.3% for α-tocopherol) and for retinol 3.7%. Serum cholesterol was analyzed by a standard enzymatic method at RIVM using an auto-analyzer (LX-20 Pro, Beckman-Coulter, Woerden, The Netherlands).

### 2.4. Statistical Analyses

Demographic characteristics were described using means and SD for continuous variables (age, weight, body mass index (BMI (kg/m^2^))) and frequencies (%) for categorical variables (gender, smoking status, age groups, and country). Differences in characteristics between age groups and dietary habits were compared by one-way ANOVA (continuous variables) and Pearson’s chi-squared test (prevalence).

Data on nutritional plasma biomarkers were transformed to achieve normal distribution using square root (SR) or logarithmic (LN) transformation as appropriate and are described by geometric means with 95% confidence intervals (CI). The association of each biomarker with age was assessed by linear regression analysis (Pearson product-moment correlation coefficient *r*). Multiple linear regression models with a forward stepwise approach were applied to identify independent plasma biomarkers with the highest correlation with age; all biomarkers were included and only those with a statistically significant high correlation (*p* < 0.001) were retained in the final models. Age as a determinant (five-year age groups) of those biomarkers with highest correlations (lycopene, α-tocopherol, α-carotene, β-cryptoxanthin) was finally assessed and confirmed using general linear models adjusted for season of blood collection, country, characteristics (gender, BMI, smoking status), serum cholesterol, dietary intake (fruit, vegetables, juice, French fries, and meat), and use of vitamin supplements; partial Eta squared (*η_p_^2^*) was applied as a measure of effect size. Statistically significant differences were considered to be present at *p* < 0.05. The greatest statistically significant differences of biomarkers with higher age are presented as box plots and scatter plots with the Cleveland’s LOWESS smoothing line. All statistical analyses were carried out using SPSS software (SPSS Inc., Chicago, IL, USA; Version 11.5).

## 3. Results

A total of 2118 female and male subjects from six different European countries with a mean age of 55 (range 35–74) years were studied for demographic characteristics, dietary habits, and plasma carotenoids, tocopherols, and retinol (Table 1). Subjects in the higher age groups were shorter (*p* < 0.001), while weight was similar throughout the age groups; thus, the mean BMI as well as the prevalence of overweight and obesity were higher in the higher age groups. Furthermore, subjects in the higher age groups were less likely to be current smokers.

The evaluation of frequencies of reported intake of fruit, vegetables, and use of vitamin supplements (assessed by chi-squared test, Table 2) revealed the following: intake of fruit (≥1 serving/day) and vegetables (≥1 serving/day) were higher in women than in men (72% vs. 56%, and 69% vs. 51%, for fruit and vegetables, respectively, both *p* < 0.001) as well as in non-smokers compared to smokers (68% vs. 47% and 62% vs. 50%, for fruit and vegetables, respectively, both *p* < 0.001). The same is true for the frequencies of reported use of vitamin supplements (≥1 supplement/week) which were also higher in women than in men (22% vs. 17%, *p* = 0.002) and in non-smokers compared to smokers (21% vs. 15%, *p* = 0.019).

The daily consumption of fruit and juice were higher and the weekly meat consumption was lower with higher age. The frequencies of reported use of vitamin supplements (≥1 supplement/week) differed between countries (*p* < 0.001), being high in Germany (24%), Poland (23%), and Austria (23%) followed by Belgium (19%), Italy (15%), and Greece (14%). Also, frequencies of reported dietary intake differed between countries (*p* < 0.001): Fruit consumption was high in Italy and in Belgium where 78% and 66% of subjects, respectively, reported an intake of ≥1 serving of fruit/day; subjects from these countries also reported a high intake of vegetables (80% from Italy and 85% from Belgium with ≥1 serving of vegetables/day); French fries were frequently eaten in Belgium (40% of subjects reporting ≥1 serving/week) and Greece (26% of subjects with ≥1 serving/week), and meat consumption (≥1 serving/day) was high in Poland (30% of subjects) and Belgium (22% of subjects).

Lycopene showed the highest mean concentrations of plasma carotenoids, and except for β-carotene, all carotenoids, tocopherols, and retinol were statistically significantly associated with age (Table 3). Lycopene and α-carotene were inversely correlated with age, whereas β-cryptoxanthin, lutein, zeaxanthin, α-/γ-tocopherol, and retinol were positively correlated with age (Table 3).

These associations were confirmed in multivariable regression models (Table 4), which revealed inverse associations of age with lycopene (*r* = −0.248) and α-carotene (*r* = −0.112), and positive associations with α-tocopherol (*r* = 0.208), and β-cryptoxanthin (*r* = 0.125) (all *p* < 0.001). Cholesterol was positively associated with all carotenoids, tocopherols, and retinol (all *p* < 0.001), but was not correlated with age when simultaneously assessed as a covariate with biomarkers in the forward regression model. The multiple regression model (forward approach) with cholesterol-adjusted compounds also confirmed lycopene (*r* = −0.256), α-tocopherol (*r* = 0.157), α-carotene (*r* = −0.128), and β-cryptoxanthin (*r* = 0.117) as those markers with a statistically significant association with age (Table 4) in our study population.

**Figure 1:**
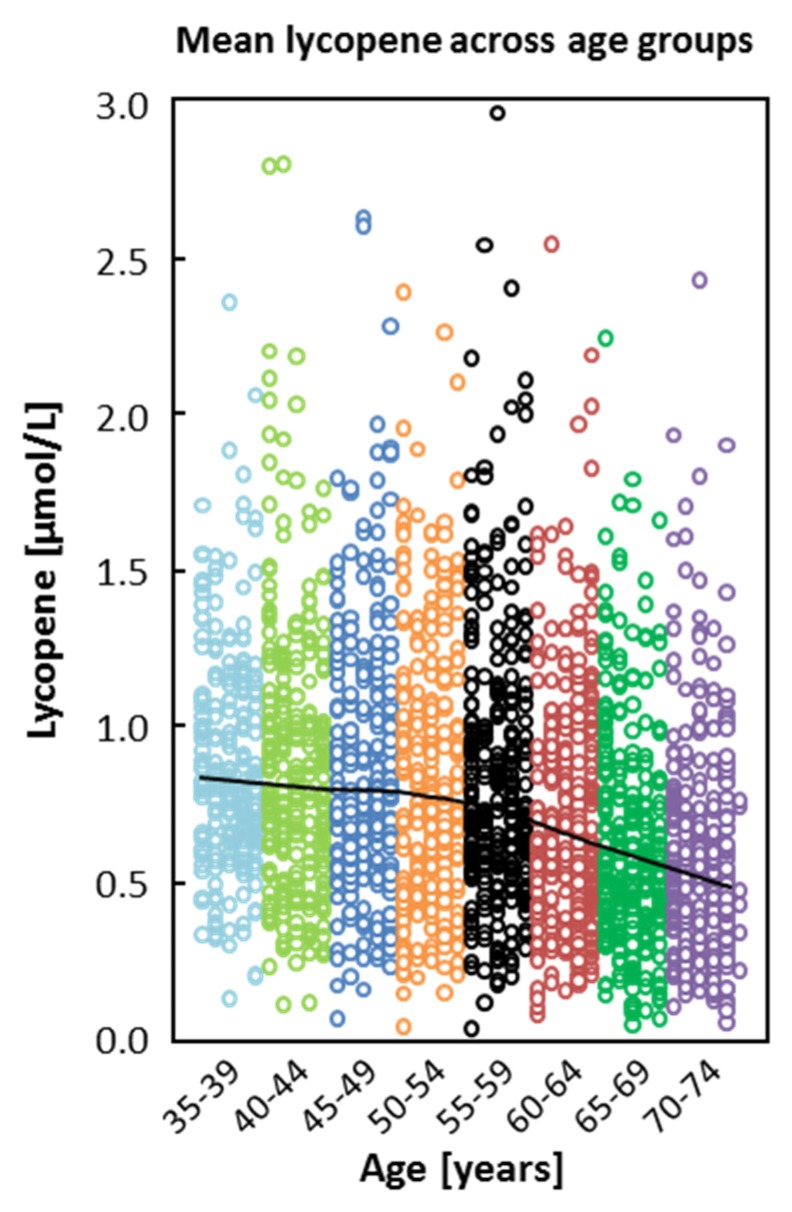
Mean lycopene across age groups. The black line indicates the mean plasma concentration of lycopene in the different age groups (*n* = 2118).

**Figure 2:**
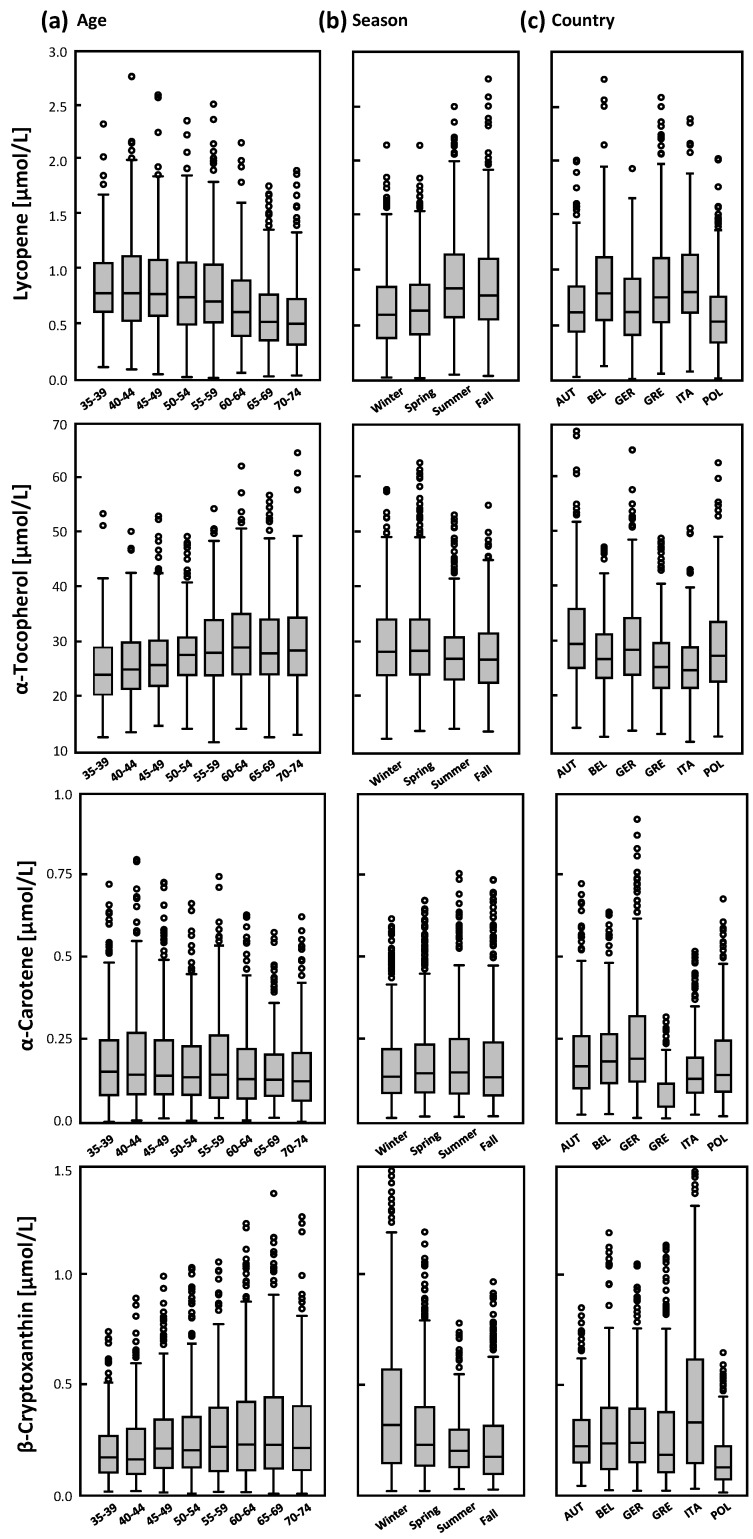
Micronutrient concentrations by age, season, and country. Box-plots of plasma lycopene, α-tocopherol, α-carotene, and β-cryptoxanthin by (**a**) age in five-year age groups; (**b**) season; and (**c**) country (AUT, Austria; BEL, Belgium; GER, Germany; GRE, Greece; ITA, Italy; POL, Poland). Extreme values are included in analyses but not shown in the figure.

Lycopene, α-tocopherol, β-cryptoxanthin, and α-carotene were predicted by age group, country (study center), season, and cholesterol (Table A1 and Figure 1). In addition, gender, smoking status, BMI, and dietary habits were statistically significantly associated with these plasma biomarkers. The associations of age with lycopene and α-tocopherol are shown in Figure 1 and Figure 2. Plasma biomarkers were cross-sectionally associated with age groups, season, and country (Table A1 and Figure 2); higher mean lycopene concentrations were found in subjects from Belgium, Greece, and Italy than in subjects from the other countries, which is in agreement with the high fruit and vegetable consumption but also high intake of French fries in these countries. Regarding season, lycopene was higher in summer (and fall) and both mean α-tocopherol and β-cryptoxanthin were higher in winter (Figure 2b).

Cholesterol was highly and positively associated with α-tocopherol (*η_p_^2^* = 0.266; Table A1).

Lower plasma lycopene was associated with overweight and/or obesity, use of vitamin supplements, daily fruit and high frequency in juice consumption, never consuming French fries and seldom but also daily meat servings. The frequent consumption of fruit or vegetables (≥ 2 servings/day) was highly predictive for higher β-cryptoxanthin and α-carotene (Table A1).

The association of lower plasma lycopene with higher age (−0.046 µmol/L or 6.5%) remained after adjusting for co-factors and covariates (Table 5). The age groups also had a high impact on lycopene (*η_p_^2^* = 0.080, Table 6) as did cholesterol; male gender was positively whereas BMI was inversely associated with lycopene in the multiple linear regression model. The association of higher α-tocopherol with age remained after adjusting for country and season, and remained if all covariates were assessed (0.486 vs. 0.667 µmol/L (1.7% vs. 2.4%)) (Table 5); cholesterol explained most of variance in α-tocopherol (*η_p_^2^* = 0.336), followed by age group, vitamin supplement use, and juice consumption (Table 6). The inverse association of α-carotene with age remained statistically significant even after adjusting for all covariates (Table 5); cholesterol, vegetable and fruit consumption, and female gender were positively while BMI, smoking, and age group were inversely associated with plasma α-carotene in the multiple regression model (Table 6).

## 4. Discussion

The present study demonstrates that plasma carotenoids, tocopherol, and retinol are related to age in a healthy European age-stratified population. For lycopene, α-tocopherol, β-cryptoxanthin, and α-carotene these associations remained even after adjusting for cholesterol, BMI, dietary habits (intake of fruit, vegetables, juice, and meat), use of vitamin supplements, gender, smoking status, country, and season. The reported higher frequency of vitamin supplement use, and fruit and vegetable intake among females and non-smokers is consistent with statistically significantly higher plasma α-tocopherol, β-cryptoxanthin, and α-carotene. Higher fruit and vegetable intake with corresponding higher plasma carotenoids in females than in males was previously reported in younger and elderly populations [25,26,27]. Because the intake of fruit is related to higher plasma β-cryptoxanthin, frequent intake of fruit in the higher age groups most likely contributed to higher β-cryptoxanthin with age. The main dietary sources of β-cryptoxanthin are orange fruits and their products, e.g., juice [28]. However, the season and thus the availability of fruit and vegetables as well as differences in dietary habits between countries are important predictors of plasma carotenoids; season and country are also known to have a strong influence on plasma carotenoids [11,12,15,29].

Associations of individual plasma carotenoids and tocopherols with age have previously been described [16,17,18,30]. Our results confirm those of a small sub-cohort (*n* = 272) of the U.S. Health Professional Follow-up and the Nurses’ Health Study, which revealed that plasma lycopene was inversely correlated with age in men and β-cryptoxanthin was positively associated with age in women (mean age: 54 years) [16]. Age was positively associated with serum concentrations and dietary intake of all carotenoids except with lycopene in the Nutritional Factors in Eye Disease Study (mean age 65 ± 9 years) [17]. Lycopene was inversely associated with age and the only carotenoid showing a statistically significant correlation with age after adjusting for gender, smoking status, BMI, supplement use, and serum cholesterol [17], in agreement with our results. In our study not only lycopene but also α-carotene was negatively associated with age. For α-carotene our study is the first to show statistically significantly lower plasma α-carotene concentrations with higher age in an age-stratified group of men and women; however, the reason for the lower α-carotene concentrations cannot be explained by our data on dietary habits. Since plasma α-carotene is reduced in the higher age groups in both genders, the lower α-carotene concentration in men compared to women (Table 6 and Table A1) does not explain this finding. Two studies showing a statistically significant positive association of plasma α-carotene with age, carried out in the United States [17] and in Venezuela [18], do not show an altered dietary intake in the higher age groups and are therefore not able to explain the higher plasma α-carotene concentrations with a higher consumption of fruit/vegetables in these individuals.

The study in Venezuela, among 35–69-year-old women (*n* = 718) and men (*n* = 646), also showed higher plasma β-carotene and β-cryptoxanthin with higher age among women, but lower plasma lycopene with higher age in both men and women [18]; the frequency of fruit and vegetable intake was statistically significantly associated with plasma lycopene. In the Framingham Heart Study, lycopene concentration, in contrast to all other carotenoids, was lower with age (as an independent factor besides BMI, cholesterol, smoking, and dietary intake of fruit, vegetables, juice, French fries, and meat) and use of vitamin supplements, and was lowest among subjects >85 years [30]. Dietary carotene intake and plasma α-tocopherol, β-cryptoxanthin, and α-/β-carotene were lower in men (*n* = 230; 67–96 years) than in women (*n* = 408; 68–94 years) [30]. In a sub-sample (*n* = 2969) within the European multicenter study EPIC following an age-stratified sampling schema (five-year groups; 45–64 years) plasma lycopene was lower with age in both men and women, and higher in summer when tomatoes are available [11]. All these previous studies and our results point to lower plasma lycopene concentrations with higher age.

The question arising is whether (a) changes in diet; (b) reduced bioavailability; or (c) different storage patterns in various organs during aging are responsible for the changes observed in older subjects in the present study.

Various factors influence the circulating concentrations and bioavailability of micronutrients, including lifestyle, metabolism, energy intake, food preparation, fat intake (plasma lipid concentration), and interactions between nutrients and drugs [31,32]. In the NHANES study with 3413 participants aged 17–90 years, race, supplement use, alcohol consumption, BMI, blood pressure, and consumption of non-tomato vegetables, fruit, and juices were not associated with serum lycopene, whereas age, gender, region, smoking, serum cholesterol and triacylglycerol, and dietary intake of fat, tomatoes, pizza, and pasta were statistically significant determinants of serum lycopene [33]. In fact, multivariate-adjusted serum lycopene concentrations (adjusted for sex, geographical location, race/ethnicity, age, education, poverty income ratio, vitamin/mineral supplement use, alcohol consumption, serum cotinine, BMI, serum total cholesterol, serum triacylglycerol, and dietary intake of fat, tomatoes, non-tomato vegetables, fruit and juices, pizza, and pasta as categorical independent covariates) were 48.3% lower in people over 70 years than in those younger than 30 years (*p* < 0.0001) [33].

An age-related decrease in lycopene absorption resulting in reduced lycopene concentrations in older subjects has been previously observed [33,34]. Since lycopene is absorbed primarily from tomatoes and tomato products, it is plausible that specific groups (e.g., men, younger subjects, subjects from Italy or Belgium), who consume relatively more tomato-based products (e.g., ketchup and tomato sauce) have higher plasma lycopene than other groups. Other studies demonstrated that older persons were likely to consume less dietary fat and fewer processed tomato products, which were stated to cause heartburn [33,34]. In terms of bioavailability, only lycopene but not α-carotene, β-carotene, and lutein showed a statistically significantly lower response (−40%, *p* < 0.04), as demonstrated in old compared to young subjects [34], implying that the bioavailability of lycopene in the elderly is impaired. This effect on the carotenoid bioavailability appeared to be independent of the food matrix (tomato) because the standardized β-carotene response for a tomato-containing meal did not differ between the two groups [34].

The bioavailability of lycopene could be, unlike other carotenoids, more dependent on age-related changes such as the physicochemical conditions of the gastrointestinal tract [35,36] in view of the fact that the absorption efficiency of lycopene is very low [37]. The combination of physiological, behavioral, and lifestyle factors is most likely responsible for the different plasma carotenoid concentrations [17].

Nevertheless, storage effects need to be considered, especially in obese persons, where a weak correlation between intake and concentrations of carotenoids in adipose tissues was observed [38]. Carotenoids are lipophilic substances stored in the adipose tissue, which could explain their inverse association with BMI [17,39,40]. Serum α-/β-carotene, β-cryptoxanthin, and lutein/zeaxanthin but not lycopene were inversely associated with BMI [17]. This change in serum carotenoids (over seven years) was inversely associated with the change in BMI among young non-smokers (25 ± 3.6 years, 45% male, 56% white) from the CARDIA study [39]. The authors also suggest that the oxidative stress associated with higher BMI may have caused lower carotenoid concentrations. Serum β-carotene was negatively associated with both general and central adiposity, whereas serum α-tocopherol was positively associated with central adiposity [40].

The limitations of the study include the rather basic information on dietary habits. For instance, only the self-reported frequency (given in ‘per week’ or ‘per day’) of consumption for glasses of juice (not specified which kind), supplement use (not specified), meat consumption, French fries, fruit, and vegetables were collected. Another limitation is that the questionnaires did not ask for individual food items within the food groups (i.e., tomatoes or oranges). This information might have strengthened our suggestions of independent associations of micronutrients with age. We focused mainly on those food groups that are relevant regarding associations of plasma concentrations with age, i.e., plasma β-cryptoxanthin and fruit, or lycopene and vegetables. The cross-sectional design may reflect only occasional dietary intake; nevertheless, knowledge of the season partly corrects for the influence of a different availability of fruit and vegetables and therefore plasma carotenoid concentrations in the multivariable models. The assessment of dietary intake was not the main focus of the project but did give valuable information on dietary habits that correlate well with the parameters analyzed in plasma samples. The questionnaires were standardized, meaning that one questionnaire was uniformly used for all subjects recruited. It was drafted in English and translated into each national language. The results from our study confirm that persons with higher intakes of fruit or vegetables have higher levels of plasma β-cryptoxanthin and α-carotene. Whether this association persists into later life remains to be assessed in specific studies involving a higher number of older individuals.

The continuous age distribution from 35 to 74 years and the equal number of men and women included in the study are a feature distinguishing this study from others. Furthermore, the samples of this large European multicenter study were collected from fasting subjects. Potential confounding factors were assessed, and samples were distributed in a blinded manner to guarantee unbiased measurement and interpretation. The analyses of plasma micronutrients as well as cholesterol were carried out in a single laboratory each, with internal quality controls so that inter-laboratory variation can be excluded.

Our models in the present study suggest an independent inverse association of α-carotene and lycopene with age because these associations remained in multivariate models adjusted for multiple covariates including dietary habits (intake of fruit, vegetables, juice, and meat, and vitamin supplement use).

We conclude for the present study that age was inversely associated with lycopene and α-carotene but positively with α-tocopherol in both women and men from different European countries. These relations hold true after adjusting for well-known factors affecting plasma concentrations of carotenoids and tocopherols such as season, country, smoking status, gender, use of vitamin supplements, and intake of fruit, and vegetables. The lower plasma lycopene and α-carotene with age may be due to a combination of several effects including change of dietary habits, lifestyle, impaired bioavailability of nutrients, storage pattern, and/or increased demand of antioxidants with age.

## Figures and Tables

**Table 1:** Characteristics of the study population by age groups (*n* = 2118).

Age group (years)	35–74	35–39	40–44	45–49	50–54	55–59	60–64	65–69	70–74	
*n*	2118	228	244	265	276	273	289	268	275	*p*
Age (years) ^1^	55.1 ± 11	37.1 ± 1.4	41.9 ± 1.5	47.1 ± 1.4	51.9 ± 1.4	56.9 ± 1.5	62.1 ± 1.4	66.8 ± 1.4	72.2 ± 1.5	<0.001
Male (%, (*n*))	49 (1042)	49 (111)	47 (116)	47 (125)	49 (134)	49 (133)	54 (155)	50 (133)	49 (135)	0.876
Smoker, current (%, (*n*))	19 (402)	23 (53)	23 (57)	24 (64)	22 (60)	24 (65)	17 (50)	9 (24)	10 (29)	
Smoker, former (%, (*n*))	31 (654)	23 (52)	17 (42)	28 (74)	34 (93)	30 (83)	42 (120)	34 (92)	36 (98)	<0.001
Smoker, never (%, (*n*))	50 (1062)	54 (123)	60 (145)	48 (127)	44 (123)	46 (125)	41 (119)	57 (152)	54 (148)	
Height (cm) ^1^	169 ± 9.5	172 ± 10	171 ± 10	171 ± 9.4	170 ± 8.8	168 ± 9.1	168 ± 8.9	167 ± 9.2	166 ± 8.9	<0.001
Weight (kg) ^1^	75 ± 15	74 ± 17	74 ± 15	74 ± 15	75 ± 15	75 ± 16	76 ± 15	76 ± 13	75 ± 12	0.594
BMI (kg/m^2^) ^1^	26.1 ± 4.5	24.8 ± 4.3	25.2 ± 4.9	25.3 ± 4.0	26.0 ± 4.6	26.4 ± 4.6	26.9 ± 4.5	27.2 ± 4.3	27.0 ± 4.0	<0.001
<25 (%, (*n*))	45 (956)	57 (131)	57 (138)	52 (139)	48 (132)	45 (123)	37 (108)	34 (92)	34 (93)	
25 to <30 (%, (*n*))	38 (802)	29 (65)	32 (79)	37 (96)	35 (97)	36 (99)	42 (120)	46 (124)	44 (122)	<0.001
≥30 (%, (*n*))	17 (360)	14 (32)	11 (27)	11 (30)	17 (47)	19 (51)	21 (61)	20 (52)	22 (59)	
Austria (%, (*n*))	18 (384)	21 (48)	21 (50)	19 (50)	17 (47)	17 (46)	17 (50)	18 (48)	16 (45)	
Belgium (%, (*n*))	12 (255)	6 (13)	7 (18)	12 (31)	14 (38)	14 (39)	14 (40)	13 (36)	14 (40)	
Germany (%, (*n*))	16 (346)	11 (26)	14 (35)	18 (48)	17 (46)	17 (47)	17 (50)	18 (47)	17 (47)	
Greece (%, (*n*))	18 (379)	19 (43)	21 (50)	17 (46)	19 (53)	16 (44)	17 (48)	16 (44)	19 (51)	0.518
Italy (%, (*n*))	18 (383)	22 (49)	20 (49)	18 (48)	18 (50)	18 (48)	16 (47)	18 (48)	16 (44)	
Poland (%, (*n*))	18 (371)	21 (49)	17 (42)	16 (42)	15 (42)	18 (49)	19 (54)	17 (45)	18 (48)	

^1^ Values are means ± SD; *p*-value: One-way ANOVA (continuous variables) and Pearson’s chi-squared test (prevalence).

**Table 2:** Frequency of reported intake of food groups and use of vitamin supplements by age group, country, gender, smoking status, and BMI ^1^.

	Vitamin Supplements	Fruit	Vegetables	Juice	French Fries	Meat	Meat
	≥1 Suppl./Week	≥1 Serv./Day	≥1 Serv./Day	≥1 Glass/Day	≥1 Serv./Week	≤1 Serv./Week	≥7 Serv./Week
All (*n* = 2118)	20 (415)	64 (1363)	60 (1270)	32 (675)	17 (361)	9 (201)	12 (250)
**Age Group, *χ*^2^**	0.369	<0.001	0.142	0.041	0.433	0.047	0.025
35–39 years (*n* = 228)	20 (46)	51 (116)	55 (126)	41 (93)	20 (45)	6 (13)	18 (42)
40–44 years (*n* = 244)	20 (48)	53 (129)	57 (138)	35 (86)	20 (50)	8 (20)	13 (31)
45–49 years (*n* = 265)	17 (44)	59 (156)	57 (151)	34 (90)	16 (42)	9 (25)	9 (25)
50–54 years (*n* = 276)	20 (56)	60 (165)	58 (160)	29 (80)	17 (48)	6 (18)	10 (28)
55–59 years (*n* = 273)	18 (50)	67 (182)	60 (163)	29 (78)	18 (49)	10 (28)	10 (27)
60–64 years (*n* = 289)	23 (67)	73 (210)	65 (189)	29 (84)	16 (46)	12 (36)	9 (26)
65–69 years (*n* = 268)	16 (43)	73 (195)	62 (166)	31 (84)	13 (35)	13 (35)	13 (34)
70–74 years (*n* = 275)	22 (61)	76 (210)	64 (177)	29 (80)	17 (46)	9 (26)	13 (37)
**Country, *χ*^2^**	<0.001	<0.001	<0.001	<0.001	<0.001	<0.001	<0.001
Austria (*n* = 384)	23 (89)	60 (230)	42 (161)	47 (179)	18 (69)	12 (45)	2 (9)
Belgium (*n* = 255)	19 (49)	66 (167)	85 (217)	35 (89)	40 (101)	3 (9)	22 (55)
Germany (*n* = 346)	24 (82)	67 (232)	46 (158)	45 (155)	3 (11)	17 (59)	4 (13)
Greece (*n* = 379)	14 (52)	57 (218)	50 (189)	24 (92)	26 (98)	14 (55)	5 (18)
Italy (*n* = 383)	15 (56)	78 (298)	80 (305)	14 (53)	8 (32)	7 (26)	11 (43)
Poland (*n* = 371)	23 (87)	59 (218)	65 (240)	29 (107)	13 (50)	2 (7)	30 (112)
**Gender, *χ*^2^**	0.002	<0.001	<0.001	0.051	<0.001	<0.001	0.002
Male (*n* = 1042)	17 (176)	56 (585)	51 (527)	34 (353)	21 (224) **	7 (73) **	14 (146) *
Female (*n* = 1076)	22 (239) *	72 (778) **	69 (743) **	30 (322)	13 (137)	12 (128)	10 (104)
**Smoking, *χ*^2^**	0.019	<0.001	<0.001	0.150	0.001	0.872	0.142
Yes (*n* = 402)	15 (62)	47 (188)	50 (199)	29 (116)	23 (91) *	10 (39)	14 (56)
No (*n* = 1716)	21 (353) *	68 (1175) **	62 (1071) **	33 (559)	16 (270)	9 (162)	11 (194)
**BMI, *χ*^2^**	0.004	0.059	0.020	<0.001	0.391	<0.001	<0.001
<25 (*n* = 956)	23 (217)	67 (641)	63 (600)	36 (340)	17 (160)	12 (117)	9 (64)
25 to <30 (*n* = 802)	17 (140)	62 (496)	56 (451)	32 (258)	16 (131)	8 (61)	12 (99)
≥30 (*n* = 359)	16 (58)	63 (225)	61 (218)	21 (77)	19 (70)	6 (23)	19 (67)

^1^ Values are percentage (numbers); suppl., use of vitamin supplement; serv., servings; *χ*^2^: Pearson’s chi-squared test (* *p* < 0.05; ** *p* < 0.001).

**Table 3:** Concentrations of carotenoids, tocopherols, retinol, and cholesterol and their correlation with age (*n* = 2118).

Compound ^1^	Geometric Mean	Percentiles		Age Correlation	
		2.5	97.5	*r*	*p*
Lycopene	0.701	0.153	1.748	−0.239	<0.001
β-Cryptoxanthin	0.203	0.033	1.064	0.121	<0.001
α-Carotene	0.139	0.029	0.643	−0.090	<0.001
Lutein	0.276	0.075	0.672	0.077	<0.001
Zeaxanthin	0.045	0.007	0.135	0.063	0.004
β-Carotene	0.540	0.133	1.856	−0.042	0.051
α-Tocopherol	27.8	17.5	48.5	0.203	<0.001
γ-Tocopherol	1.29	0.400	3.55	0.119	<0.001
Retinol	1.71	0.980	2.73	0.121	<0.001
Cholesterol	5.55	3.68	7.79	0.081	<0.001

^1^ All compounds in µmol/L, except cholesterol (*n* = 1993) given in mmol/L. Pearson’s correlation *r* with age (years).

**Table 4:** Association of carotenoids and α-tocopherol with age.

	(B)	95% CI	Partial *R*	(*R^2^*)	*p*
(µmol/L) ^1^	51.35	49.36, 53.34			<0.001
Lycopene	−6.638	−7.778, −5.499	−0.248	0.059	<0.001
α-Tocopherol	0.289	0.229, 0.348	0.208	0.042	<0.001
α-Carotene	−5.761	−8.012, −3.524	−0.112	0.011	<0.001
β-Cryptoxanthin	5.062	3.302, 6.823	0.125	0.011	<0.001
(µmol/mmol) ^2^	53.03	50.64, 55.41			<0.001
Lycopene/Cholesterol	−39.15	−45.64, −32.65	−0.256	0.076	<0.001
α-Tocopherol/Cholesterol	1.394	1.009, 1.780	0.157	0.022	<0.001
α-Carotene/Cholesterol	−36.32	−48.68, −23.96	−0.128	0.011	<0.001
β-Cryptoxanthin/Cholesterol	25.85	16.21, 35.49	0.117	0.012	<0.001

^1^ Multiple regression analyses (forward stepwise approach) with age (years) as the dependent variable; all measured biomarkers including cholesterol were assessed as covariates in the initial model (*n* = 2118), sum *R* = 0.350, sum *R^2^* = 0.123; ^2^ Biomarkers adjusted for cholesterol (µmol/mmol) were assessed in the initial model (*n* = 1993); sum *R* = 0.348, sum *R^2^* = 0.121. Regression coefficient *B* represents higher (+)/lower (−) age (years) with each unit (µmol/L or µmol/mmol) increase in respective compounds, e.g., per 1 µmol/L increase in lycopene, mean age was 6.6 years lower (−6.638 years).

**Table 5:** Association of carotenoids and α-tocopherol with age groups after adjusting for covariables.

	*B*	95% CI	*p*	(*η*^2^)	Difference (µmol/L) ^1^	Difference (%) ^2^
(SR) Lycopene						
Age groups ^3^	−0.028	−0.032, −0.023	<0.001	0.068	−0.047	-6.7
Age groups ^4^	−0.026	−0.030, −0.022	<0.001	0.070	−0.044	-6.3
Age groups ^5^	−0.027	−0.031, −0.023	<0.001	0.080	−0.046	-6.5
(LN) α-Tocopherol						
Age groups ^3^	0.024	0.019, 0.029	<0.001	0.044	0.667	2.4
Age groups ^4^	0.023	0.018, 0.028	<0.001	0.041	0.636	2.3
Age groups ^5^	0.018	0.014, 0.022	<0.001	0.035	0.486	1.7
(LN) α-Carotene						
Age groups ^3^	−0.038	−0.053, −0.023	<0.001	0.012	−0.005	-3.8
Age groups ^4^	−0.045	−0.059, −0.031	<0.001	0.018	−0.006	-4.4
Age groups ^5^	−0.050	−0.063, −0.037	<0.001	0.029	−0.007	-4.8
(LN) β-Cryptoxanthin						
Age groups ^3^	0.036	0.019, 0.053	<0.001	0.008	0.007	3.6
Age groups ^4^	0.031	0.015, 0.047	<0.001	0.007	0.006	3.0
Age groups ^5^	0.012	−0.002, 0.027	0.102	0.001	0.002	1.1

^1^ Mean difference per age group of back-transformed data (µmol/L) considering the intercept of each model; ^2^ Differences in biomarker concentrations as a percentage (%) of the geometric means (lycopene: 0.701 µmol/L; α-tocopherol: 27.85 µmol/L; β-cryptoxanthin: 0.203 µmol/L; α-carotene: 0.139 µmol/L); ^3^ Linear regression analysis with age groups (*n* = 8) as a covariate; ^4^ Multiple linear regression with age groups (*n* = 8) as a covariate and country and season as co-factors; ^5^ Multiple linear regression with age group, cholesterol, BMI, frequency of dietary habits (fruit, vegetables, juice, and meat per week), use of vitamin supplements, gender, and smoking status as covariates, and country and season as co-factors. (SR) square root transformed; (LN) logarithmic transformed. Regression coefficient *B* represents the increase/decrease in the respective compound for each multiple linear regression model.

**Table 6:** Associations of age, demographic characteristics, and dietary habits with plasma carotenoids and α-tocopherol (µmol/L) ^1^.

	(SR) Lycopene			(LN) α-Tocopherol			(LN) α-Carotene			(LN) β-Cryptoxanthin		
	*B*	*p*	*η*^2^	*B*	*p*	*η*^2^	*B*	*p*	*η*^2^	*B*	*p*	*η*^2^
Age group (1–8)	−0.027	<0.001	0.080	0.018	<0.001	0.035	−0.050	<0.001	0.029	0.012	0.102	0.001
Cholesterol (mmol/L)	0.068	<0.001	0.109	0.138	<0.001	0.336	0.118	<0.001	0.036	0.105	<0.001	0.023
BMI (kg/m^2^)	−0.006	<0.001	0.015	0.002	0.054	0.002	−0.045	<0.001	0.086	−0.024	<0.001	0.021
Gender (male = 1)	0.034	<0.001	0.007	−0.004	0.704	0.000	−0.144	<0.001	0.012	−0.239	<0.001	0.025
Smoking (yes = 1)	−0.015	0.197	0.001	0.016	0.166	0.001	−0.336	<0.001	0.040	−0.325	<0.001	0.030
Vitamin suppl. (*n*/week)	−0.003	0.078	0.002	0.012	<0.001	0.023	0.008	0.157	0.001	0.001	0.895	0.000
Fruit (*n*/week)	−0.001	0.405	0.000	0.000	0.717	0.000	0.023	<0.001	0.018	0.067	<0.001	0.107
Vegetables (*n*/week)	0.002	0.189	0.001	0.001	0.476	0.000	0.041	<0.001	0.034	0.000	0.958	0.000
Juice (*n*/week)	−0.001	0.338	0.000	0.002	0.015	0.003	−0.001	0.557	0.000	0.006	0.014	0.003
French Fries (*n*/week)	0.005	0.260	0.001	−0.002	0.601	0.000	−0.015	0.299	0.001	−0.032	0.041	0.002
Meat (*n*/week)	−0.001	0.723	0.000	0.000	0.927	0.000	−0.006	0.405	0.000	−0.003	0.679	0.000

^1^ Multiple linear regression model, adjusted for country and season. (SR) square root transformed; (LN) logarithmic transformed. Regression coefficient *B* represents the increase/decrease in compound with each unit increase in respective factor/variable.

## Appendix A

**Table A1:** Associations of age and demographic characteristics with plasma lycopene, α-tocopherol, β-cryptoxanthin, and α-carotene.

		Lycopene		α-Tocopherol		α-Carotene		β-Cryptoxanthin	
Factors	*n*	*η_p_^2^*, GM	95% CI	*η_p_^2^*, GM	95% CI	*η_p_^2^*, GM	95% CI	*η_p_^2^*, GM	95% CI
**Age group (years)**		*0.079 ***		*0.051 ***		*0.013 ***		*0.016 ***	
35–39	228	0.810 ^a^	0.757, 0.865	25.1 ^c^	24.3, 25.9	0.153 ^a,b^	0.138, 0.170	0.167 ^c^	0.148, 0.187
40–44	244	0.808 ^a^	0.757, 0.861	25.7 ^c^	24.9, 26.5	0.155 ^a^	0.140, 0.171	0.168 ^b,c^	0.151, 0.188
45–49	265	0.803 ^a^	0.755, 0.854	26.7 ^c,b^	25.8, 27.5	0.155 ^a^	0.141, 0.170	0.209 ^a–c^	0.187, 0.232
50–54	276	0.759 ^a^	0.712, 0.806	28.1 ^b,a^	27.2, 28.9	0.141 ^a–c^	0.128, 0.155	0.213 ^a–c^	0.192, 0.236
55–59	273	0.766 ^a^	0.719, 0.814	28.9 ^a^	28.1, 29.8	0.142 ^a–c^	0.129, 0.156	0.214 ^a,b^	0.192, 0.238
60–64	289	0.650 ^b^	0.609, 0.692	29.6 ^a^	28.7, 30.5	0.132 ^a–c^	0.120, 0.144	0.215 ^a,b^	0.194, 0.238
65–69	268	0.559 ^b,c^	0.520, 0.600	29.1 ^a^	28.3, 30.1	0.124 ^b,c^	0.113, 0.136	0.238 ^a^	0.214, 0.264
70–74	275	0.521 ^c^	0.483, 0.561	29.3 ^a^	28.4, 30.2	0.122 ^c^	0.111, 0.134	0.202 ^a–c^	0.182, 0.224
**Country**		*0.082 ***		*0.068 ***		*0.166 ***		*0.114 ***	
Austria	384	0.630 ^b^	0.598, 0.663	30.8 ^a^	30.0, 31.5	0.164 ^b^	0.153, 0.177	0.220 ^b^	0.202, 0.240
Belgium	255	0.807 ^a^	0.754, 0.861	27.8 ^b^	26.9, 28.7	0.174 ^a,b^	0.159, 0.190	0.210 ^b^	0.190, 0.233
Germany	346	0.640 ^b^	0.603, 0.678	29.4 ^a,b^	28.6, 30.1	0.198 ^a^	0.183, 0.213	0.228 ^b^	0.208, 0.249
Greece	379	0.802 ^a^	0.755, 0.849	25.9 ^c^	25.3, 26.6	0.073 ^d^	0.068, 0.079	0.190 ^b^	0.175, 0.207
Italy	383	0.841 ^a^	0.802, 0.881	25.5 ^c^	24.8, 26.1	0.133 ^c^	0.123, 0.143	0.305 ^a^	0.280, 0.332
Poland	371	0.539 ^c^	0.506, 0.573	28.3 ^b^	27.6, 29.0	0.147 ^b,c^	0.137, 0.159	0.115 ^c^	0.106, 0.125
**Gender**		*0.002*		*0.005 **		*0.051 ***		*0.055 ***	
Male	1042	0.717	0.694, 0.743	27.3 ^b^	26.9, 27.8	0.116 ^b^	0.111, 0.122	0.164 ^b^	0.156, 0.173
Female	1076	0.685	0.662, 0.709	28.4 ^a^	27.9, 28.8	0.166 ^a^	0.159, 0.174	0.250 ^a^	0.237, 0.263
**Season**		*0.067 ***		*0.014 ***		*0.002*		*0.043 ***	
Winter	499	0.594 ^b^	0.564, 0.623	28.9 ^a^	27.7, 29.1	0.133	0.124, 0.143	0.266 ^a^	0.246, 0.287
Spring	675	0.627 ^b^	0.599, 0.654	28.8 ^a^	28.3, 29.4	0.142	0.134, 0.153	0.215 ^b^	0.202, 0.230
Summer	496	0.841 ^a^	0.805, 0.878	26.8 ^b^	26.2, 27.4	0.144	0.135, 0.155	0.176 ^c^	0.163, 0.190
Fall	448	0.794 ^a^	0.757, 0.834	27.0 ^b^	26.4, 27.7	0.136	0.126, 0.146	0.161 ^c^	0.148, 0.174
**Cholesterol**		*0.044 ***		*0.266 ***		*0.027 ***		*0.024 ***	
Low Tertile	664	0.604 ^c^	0.577, 0.632	23.7 ^c^	23.3, 24.1	0.120 ^c^	0.113, 0.127	0.168 ^b^	0.157, 0.179
Mid Tertile	662	0.691 ^b^	0.663, 0.721	28.0 ^b^	27.5, 28.5	0.140 ^b^	0.131, 0.148	0.211 ^a^	0.198, 0.226
High Tertile	667	0.806 ^a^	0.776, 0.839	33.0 ^a^	32.4, 33.5	0.165 ^a^	0.156, 0.175	0.233 ^a^	0.218, 0.249
**Smoking**		*0.002*		*0.002 **		*0.071 ***		*0.058 ***	
Current smoker	402	0.735	0.694, 0.774	27.1 ^b^	26.4, 27.8	0.090 ^b^	0.083, 0.097	0.130 ^b^	0.120, 0.142
Current non-smoker	1716	0.694	0.676, 0.712	28.0 ^a^	27.7, 28.4	0.154 ^a^	0.149, 0.160	0.225 ^a^	0.216, 0.235
**BMI** (kg/m^2^)		*0.020 ***		*0.003 **		*0.104 ***		*0.021 ***	
<25	956	0.745 ^a^	0.719, 0.771	27.4 ^b^	27.0, 27.9	0.179 ^a^	0.171, 0.189	0.234 ^a^	0.221, 0.247
25 to <30	802	0.701 ^a^	0.672, 0.728	28.4 ^a^	27.9, 28.9	0.126 ^b^	0.120, 0.133	0.185 ^b^	0.174, 0.197
≥30	359	0.591 ^b^	0.553, 0.629	27.8 ^a,b^	27.0, 28.5	0.089 ^c^	0.082, 0.096	0.172 ^b^	0.157, 0.188
**Vitamin suppl.**		*0.007 **		*0.026 ***		*0.016 ***		*0.004 **	
No suppl.	1359	0.726 ^a^	0.706, 0.748	27.2 ^c^	26.8, 27.6	0.129 ^b^	0.124, 0.135	0.204 ^a,b^	0.194, 0.244
<1 suppl./day	517	0.661 ^b^	0.627, 0.694	28.3 ^b^	27.6, 28.9	0.153 ^a^	0.143, 0.164	0.190 ^b^	0.176, 0.205
≥1 suppl./day	242	0.650 ^b^	0.602, 0.699	31.1 ^a^	30.1, 32.1	0.170 ^a^	0.154, 0.188	0.232 ^a^	0.207, 0.259
**Fruit**		*0.005 **		*0.001*		*0.065 ***		*0.162 ***	
<1 serv./day	755	0.729 ^a^	0.701, 0.759	27.7	27.2, 28.3	0.109 ^a^	0.103, 0.116	0.134 ^a^	0.126, 0.142
=1 serv./day	838	0.667 ^b^	0.642, 0.694	27.8	27.3, 28.3	0.145 ^b^	0.138, 0.153	0.214 ^b^	0.203, 0.226
≥2 serv./day	525	0.716 ^a,b^	0.682, 0.752	28.2	27.6, 28.8	0.184 ^c^	0.173, 0.197	0.341 ^c^	0.318, 0.365
**Vegetables**		*0.002*		*0.002*		*0.052 ***		*0.055 ***	
<1 serv./day	848	0.689	0.633, 0.716	28.1	27.6, 28.5	0.114 ^a^	0.108, 0.120	0.167 ^a^	0.157, 0.177
=1 serv./day	947	0.698	0.672, 0.724	28.0	27.5, 28.4	0.151 ^b^	0.144, 0.159	0.209 ^b^	0.198, 0.221
≥2 serv./day	323	0.743	0.699, 0.788	27.1	26.3, 27.9	0.187 ^c^	0.172, 0.204	0.312 ^c^	0.284, 0.343
**Juice**		*0.003 **		*0.007 **		*0.010 ***		*0.003 **	
<1 serv./day	1443	0.707 ^a^	0.685, 0.728	27.5 ^a^	27.2, 27.9	0.132 ^a^	0.127, 0.138	0.196 ^a^	0.187, 0.205
=1 serv./day	393	0.722 ^a^	0.682, 0.762	28.0 ^a^	27.3, 28.7	0.162 ^b^	0.150, 0.175	0.223 ^b^	0.205, 0.244
≥2 serv./day	282	0.645 ^b^	0.600, 0.691	29.3 ^b^	28.4, 30.2	0.147 ^a,b^	0.134, 0.161	0.212 ^a,b^	0.191, 0.236
**French Fries**		*0.019 ***		*0.004 **		*0.017 ***		*0.014 ***	
no serv./week	562	0.616 ^a^	0587, 0.648	28.6 ^a^	28.0, 29.2	0.160 ^a^	0.150, 0.171	0.229 ^a^	0.213, 0.246
≤1 serv./week	1195	0.722 ^b^	0.700, 0.746	27.7 ^b^	27.3, 28.1	0.137 ^b^	0.131, 0.144	0.204 ^b^	0.194, 0.215
≥2 serv./week	361	0.766 ^b^	0.722, 0.808	27.4 ^b^	26.7, 28.1	0.117 ^c^	0.108, 0.127	0.165 ^c^	0.151, 0.181
**Meat**		*0.006 **		*0.001*		*0.002*		*0.017 ***	
≤1 serv./week	201	0.646 ^a^	0.594, 0.701	28.4	27.4, 29.5	0.152	0.136, 0.170	0.242 ^a^	0.214, 0.273
2–6 serv./week	1667	0.717 ^b^	0.699, 0.736	27.8	27.5, 28.2	0.139	0.134, 0.144	0.208 ^a^	0.199, 0.217
≥7 serv./week	250	0.640 ^a^	0.594, 0.689	27.5	26.6, 28.4	0.131	0.119, 0.145	0.151 ^b^	0.135, 0.168

Linear models (univariate) assessing impact of covariates (age group, cholesterol, BMI) and co-factors (center, season) on plasma lycopene, α-tocopherol, α-carotene, and β-cryptoxanthin. *η_p_^2^* (partial Eta squared) as an estimate of effect size (* *p* < 0.05, ** *p* < 0.001); estimates in subgroups are given as geometric means (GM) and 95% CI; pairwise multiple comparisons using Bonferroni’s CI adjustment; values not sharing superscript letters are statistically significantly different (*p* < 0.05). Suppl.: use of vitamin supplements.
